# Approaching Environmental Health Disparities and Green Spaces: An Ecosystem Services Perspective

**DOI:** 10.3390/ijerph120201952

**Published:** 2015-02-10

**Authors:** Viniece Jennings, Cassandra Johnson Gaither

**Affiliations:** USDA Forest Service, Southern Research Station, Integrating Human and Natural Systems, 320 Green Street, Athens, GA 30602, USA; E-Mail: cjohnson09@fs.fed.us

**Keywords:** environmental health disparities, ecosystem services, green space, urban health, nature, parks

## Abstract

Health disparities occur when adverse health conditions are unequal across populations due in part to gaps in wealth. These disparities continue to plague global health. Decades of research suggests that the natural environment can play a key role in sustaining the health of the public. However, the influence of the natural environment on health disparities is not well-articulated. Green spaces provide ecosystem services that are vital to public health. This paper discusses the link between green spaces and some of the nation’s leading health issues such as obesity, cardiovascular health, heat-related illness, and psychological health. These associations are discussed in terms of key demographic variables—race, ethnicity, and income. The authors also identify research gaps and recommendations for future research.

## 1. Introduction

In the United States, health disparities are unequal health outcomes related to economic, social, and/or environmental disadvantage and correlated with factors such as race or ethnicity, gender, sexual orientation, and geographic location [1]. There are myriad reasons why health disparities may exist, including factors associated with both the built and natural environment. Research suggests that green spaces (e.g., parks, forests, green ways, and gardens) can alleviate some health concerns and promote environmentally sustainable cities [2]. Green spaces may play a key role in the development of environmental health disparities across socio-demographic groups. For example, Payne-Sturges and Gee [3] developed an environmental health disparities framework that identified greenways as a potential indicator of health in minority neighborhoods, and other scholars have outlined how interactions between the natural environment [4,5,6], health behaviors, built environment, and social dynamics are key determinants in environmental health promotion [7]. Also, a number of empirical studies have established significant correlations between green spaces and human health [8,9,10,11,12,13]. Despite these findings, however, critical features of the natural environment are not fully integrated into the health disparities dialogue. This article focuses on the role that urban green spaces play in the development of health disparities across socio-demographic groups.

We synthesize recent literature that illustrates how green spaces may serve as an intervention [14] to help reduce disparities in obesity rates, cardiovascular health, heat-related illnesses, and psychological health. This focus is important given: (1) continuing health disparities based on race and ethnicity in the U.S. and (2) growing interest in achieving environmental justice (that is, the fair treatment and meaningful involvement of all people, including racial/ethnic minorities and low income persons, in environmental decision making) [15,16,17]. We include insight from fields such as environmental health, sociology, urban ecology, planning, outdoor recreation, psychology, and land change science. Studies use a range of terms to describe urban green space [18,19]. Similarly, we describe green spaces as vegetated areas, including parks, forests, gardens, or other places with herbaceous or woody vegetation.

Before discussing this literature, we clarify the linkages between urban green spaces and public health through the role of ecosystem services, which are the medium that conveys direct and indirect benefits to humans [20]. These ecosystem services are mediated through intermediary functions such as nutrient cycling and primary production [21]. Ecosystem services are categorized into four groups: provisioning (e.g., fresh water), cultural (e.g., outdoor recreation), regulating (e.g., climate mitigation), and supporting (e.g., primary production) [20,21,22]. For example, the provisioning and regulating services of “green infrastructure,” or the network of green spaces across a landscape [23], effectively reduce storm water and related non-point pollutants [24] and filter water supplies. Also, cultural services related to outdoor recreation can impart a “sense of place” to recreationists, which can enhance emotional and psychological well-being. Daniel* et al.* [22] explains how cultural services (e.g., landscape aesthetics and recreation in urban parks) play an underestimated role in health and well-being. There is abundant evidence that ecosystem degradation can affect every dimension of health [25,26], however some are weakly described [25], most arguably for racial/ethnic minorities and low income populations.

The delivery of ecosystem services from urban green spaces can vary due to geographic context (*i.e*., the larger socio-economic and political milieu), scale (e.g., extent of benefits that can be relayed), and heterogeneity (*i.e*., structural variation across space) [21]. Services can also vary according to climate [27], human values [28], and limited funding for parks [29]. Important to the present discussion is that the ecosystem services of green spaces can be irregularly distributed across urban spaces due to power differentials between socially marginal groups and middle/upper income whites [30]. Concerning the latter, a study conducted in Tampa, FL found that neighborhoods with more black people, renters, and low income residents had drastically fewer trees on public right of ways [31]. Also, Wolch* et al.* [32] asserts that green spaces are sparse or poorly maintained in the urban core of many American cities where minority and low income people concentrate. Likewise, a Milwaukee study found a negative correlation between canopy cover and household income in relation to the proportion of Hispanic residents [33]. The study also suggested that other factors (e.g., funding for maintenance, population density,* etc.*) influenced disparate access to canopy cover and implied that higher income residents reap more ecological benefits from canopy cover since these homes contain more tree coverage on their private property [33]. Similarly, field data from Miami-Dade County (Florida) indicated that white residents had more vegetative cover, tree diversity, and more energy savings from trees while black residents had the lowest scores for most factors [27]. Minority and low-income areas in Greensboro (North Carolina) have less wooded areas, suggesting that these communities may lack the health benefits from natural park features [34] .

While numerous studies in U.S. cities indicate that minority and low-income communities have less access to green spaces [31,32,33,35,36,37,38,39], some studies have not revealed such differences [40,41]. For example, studies in Baltimore (Maryland) found that access to city parks could not be explained by racial, ethnic, and socioeconomic variables [41]; specifically, predominantly black areas had better walking access to city parks, compared to mostly white communities [42]. Many of these case studies used geographical information systems, ordinary least squares or other regression models to observe that racial/ethnic minorities and low-income populations had less access to green spaces or recreational spaces [31,36,37,38]. Despite mixed observations, we maintain that there is sufficient research suggesting that inequitable access to urban green spaces is an environmental justice issue that links characteristics of the natural environment to the development of some health disparities.

We now turn to the scholarship examining how green space access may influence health disparities across socio-demographic groups. The public health community in the U.S. has been reluctant to thoroughly explore the role of the natural environment on health [19], partly because of the need for rigorous controls of various other factors and the lack of communication among disciplines [43]. This hesitancy is reflected in Kabisch* et al.*’s [44]international review of the literature on urban green spaces, which found that only four papers on the health effects of urban green spaces were published in the U.S. between January 2000 and October 2013. While they acknowledge that their review did not include articles from national reports or local planning documents, their results suggest that research on the intersection of green space and health may not be adequately addressed in the U.S. Although we review some international studies, this article primarily highlights the work on green space and health for socio-demographic groups in the United States.

## 2. Physical Health Outcomes

In some contexts, simple exposure to green spaces may improve human health [23]. In 1984, a seminal study published in *Science* indicated that post-operative recovery rates were significantly faster for patients with a view of vegetation from their hospital rooms, compared to those with a view of a brick wall [45]. Numerous other studies also suggest that active engagement with green spaces is associated with various improvements in both physical and psychological well-being [8,12,13,46,47,48,49,50]. Obesity, cardiovascular health, and heat-related conditions are the physical health concerns covered in the following discussion. 

### Obesity

The sedentary lifestyle prevalent in contemporary, industrialized societies is detrimental to human health and well-being. Physical inactivity contributes to 3.2 million annual deaths worldwide and increases the risk of obesity and cardiovascular disease [51]. Racial/ethnic minority status, place of residence, and low socioeconomic status are other factors that contribute to the high prevalence of obesity in the U.S [52]. Research indicates that youth in low-income areas, with a sedentary lifestyle, are 3.7 times more likely to be obese compared to their active counterparts in wealthier areas (adjusted prevalence of 19.8%* versus* 6.7%) [52]. In addition, people with lower levels of educational attainment and some racial/ethnic minorities have significantly higher obesity rates and are considered high priority groups for intervention [53].

The Centers for Disease Control and Prevention (CDC) considers proximity to parks, within one-half mile of a residence, as a community design feature [54] important in health promotion because physical activity in green spaces can help to reduce obesity [55,56]. In urban areas, residents of “walkable neighborhoods” (*i.e*., those with built infrastructure supporting pedestrian mobility), tend to have more access to green spaces and are generally involved in more physical activity, thus lowering obesity rates, other factors equal [57,58]. For instance, research suggests that greater use of pedestrian-friendly parks helped to reduce type-2 diabetes for Hispanic youth in Los Angeles (California) [59], lowered body weights of disadvantaged youth in Texas and Indiana [9,60] and significantly improved muscle strength in minority youth at risk of being obese [60]. More generally, West* et al.* [61] performed a cross-sectional study across some of the largest cities in the United States and observed a positive correlation between park density (*i.e*., acres of parkland as a percent of land area) and local levels of physical activity. The implication here is that higher levels of physical activity would be associated with lower obesity rates, other factors equal [61].

However, access to green space may not be sufficient for those who are inactive, do not explore the outdoors as part of their lifestyle, or do not have community programming that supports outdoor activity [62]. For instance, minority and low-income residents in Los Angeles proposed that increasing park events and sports activities are key ways to improve local parks and increase visitation [63]. Importantly, contextual factors of an urban setting, which were referenced by Escobedo* et al.* [21], for instance, public safety [64], and park proximity to pollution sources and other urban disamenities, can discourage physical activity in minority and low-income communities [65,66]. Also, studies suggest that communities with limited park access may also have few healthy food options, which is a correlate of obesity [67,68].Thus, the efficacy of green spaces in reducing obesity rates should be linked to factors in the larger social milieu, such as access to healthy foods and outdoor recreation programs, which in concert can promote greater health and well-being. 

#### Cardiovascular Health

Cardiovascular diseases are a leading cause of death in the United States. They are influenced by factors such as genetics, diet, eating habits, and physical activity. A 2013 report from the American Heart Association estimates that nearly 44% of the U.S. population (122 million people), will have some type of cardiovascular disease by 2030 [69].Addressing racial/ethnic health disparities in cardiovascular diseases continues to be a major public health challenge in the United States [70]. In 2009, premature mortality rate from cardiovascular disease was higher for blacks compared to whites (65.5* versus* 43.2) [53]. Research also indicates that racial/ethnic minorities and low-income communities have limited access to aesthetics and recreation facilities that support cardiovascular health and well-being [71,72].

In a multi-ethnic study across neighborhoods in three states, Mujahid* et al.* [73] included the presence of shade from tree cover as a factor in neighborhood walkability. They observed that neighborhoods with higher income and fewer racial/ethnic minorities were tied to more walkability, availability of healthy foods, social cohesion and safety. Overall, they found that residents with such positive neighborhood characteristics were less likely to be hypertensive, which is linked to lower instances of cardiovascular disease [73]. Thus, this study suggests that the cultural services of ecosystems (e.g., aesthetic surroundings, health effects of outdoor exercise) may help alleviate stress and hypertension, which have a negative impact on cardiovascular health.

Mitchell and Popham found that greater exposure to green space reduced mortality from circulatory disease amongst low income populations in England [74]. Also, results from a study of fifteen American states found that an increase in cardiovascular and respiratory illness was linked to tree loss [75]. Other studies observed an inverse correlation between amount of green space, stroke incidence, and cardiovascular health, respectively [8,10]. Since parks, trails, and trees support the built environment which plays a pivotal role in physical activity, enhancing urban/environmental planning to increase physical activity is a way to improve cardiovascular health [71]. Therefore, future research on improving cardiovascular health should consider the cultural services of green spaces.

#### Heat Related Conditions

Climate change may exacerbate the urban heat island effect (UHI) (the absorption and re-release of solar energy by impervious surfaces and buildings, resulting in increased temperatures in cities). Episodes of extreme heat remain a substantial cause of preventable deaths across the United States [76,77]. Heat-related illnesses were linked with an estimated $5 billion in U.S. health costs between 2000 and 2009 [78].Furthermore, heat stress can prompt physiological responses that cause the body to become more vulnerable to illness [79].

Nationally, racial/ethnic minorities experience distinct health effects from climate change [80,81], in part because they tend to concentrate in urban areas, live in neighborhoods, and work in occupations with higher exposure to heat stress [82,83]. By providing shade and undergoing the process of evapotranspiration, green spaces help decrease surface/air temperatures which is a regulating service that buffers the UHI [84] and reduces vulnerability to climate change. Research using remote sensing data shows that ecological context, such as the presence of urban forests, significantly alleviates diurnal and seasonal urban heat islands across biomes in the continental U.S. [85]. Hence people do not necessarily have to have direct contact with green spaces to benefit from the regulating service of heat mitigation. Jesdale* et al.*’s national-level study found that African-Americans, Asians and Hispanics were at least 21% more likely than non-Hispanic whites to live in areas with less tree canopy cover which reduces heat risk [86]. Also, data from the 2006 summer heat wave in California showed that Hispanics had a significant increase in hospitalizations for cardiac conditions [87]. In a related California study, Jackson and Rosenberg found that excess deaths of Hispanic agricultural workers could be attributed to occupational exposure to heat stress [88].

Harlan* et al.* [89] studied the role of vegetation on the microclimate of eight diverse neighborhoods in Phoenix, Arizona. They found that neighborhoods with higher poverty rates, more minorities (e.g., Hispanics of Mexican origin), and lower educational attainment had significantly higher scores on a human thermal comfort index- an indicator for heat vulnerability. Minority and low-income populations that were exposed to heat-induced health risks also resided in neighborhoods that were densely populated and had less green space [89]. A study in Newark and Camden, New Jersey found low-income neighborhoods were at the greatest risk for heat hazards although these neighborhoods had less space to plant trees that can mitigate the UHI effect [90]. Since green spaces can alleviate the UHI effect and reduce some health impacts of climate change [91,92,93,94], policies that address the UHI effect should include the availability of environmental resources (e.g., green spaces) to support vulnerable populations [89].

## 3. Psychological Health

Managing stress is important to offset depression and other mental health challenges. While life changes and challenges may be similar across socio-demographic groups, socially disadvantaged populations are at a higher risk of unique sources of stress related to discrimination, unemployment and other factors [95]. A study of US households found that low-income populations were more likely to express low emotional well-being and low life evaluation [96]. According to 2009 data, persons with only a high school degree had the highest suicide rates; also, American Indian/Alaskan Native and non-Hispanic blacks had the highest suicide rate amongst adolescent and young adults [53]. Studies suggest that stress and the lack of social support are detrimental to psychological health and lead to depressive symptoms and suicide [97]. Mays* et al.* [98] argue that the lack of green spaces in impoverished communities reduces the social benefits that can help residents cope with life. Other studies across multiple locations observed that racial/ethnic minorities tend to live in neighborhoods with lower aesthetic quality, which may play a role in amplifying stress that brings about depressive symptoms [99,100]. For example, Vaughan* et al*. [101] found that even though low-income areas across Kansas City had more parks, they also had more parks with quality concerns and fewer playgrounds. Consequently, these groups are vulnerable to psychological health challenges that may be improved by exposure to green spaces.

Studies in Europe have also linked green infrastructure to lower stress, positive emotions [102,103,104], increased attention capacity, and cognitive capacity [23,105]. For instance, research out of the UK observed that greater exposure to green space had an inverse relationship to the level of stress responses (salivary cortisol) for the unemployed [106]. Studies across the U.S show similar results related to coping with stress [12,49,106,107], improving resilience [108,109,110,111], enhancing self-discipline [112], and reducing symptoms of depression [99,100,113,114,115]. For example, natural features (e.g., vegetation) around homes showed a significant positive relationship with strategies to cope with stress for mothers of children in the Head Start program, suggesting that participants may find relief in aesthetic surroundings [107]. Fan* et al*. [116] analyzed responses from a community health survey and vegetation data and found that parks foster social support and indirectly mitigate stress in Chicago communities. Likewise, Miles* et al.* [113] found that the acreage of green space was significantly correlated with fewer symptoms of depression amongst residents in Miami-Dade County. Together, these studies show how the presence of green spaces can enhance psychological well-being across socio-demographic communities.

Concepts such as sense of place and place attachment involve the aesthetic, social, physical, spiritual, and psychological qualities of a location that influence one’s feelings of attachment and belonging [117]. These intangible aspects of a physical space or place can be facilitated through contact with nature [117,118]. Green infrastructure is linked with aspects of community health such as sense of place, community identity, and social capital [23,118,119]. For example, a study of two Maryland neighborhoods found that natural features and open space played a key role in community identity, sense of place, and place attachment [120]. Since physical activity can help to reduce symptoms of depression [121], exercising in green spaces can also enhance psychological well-being. Residents of walkable neighborhoods are more likely to know their neighbors and be socially engaged in their community, compared to their counterparts in car-oriented suburbs [122]. A cross-sectional study in Denver (Colorado) explored the role of neighborhood gardens on place attachment and suggested that community and home gardens were positively linked with greater neighborhood attachment compared to people who did not garden [123]. Thus, the cultural services from green spaces may revitalize a community and encourage a sense of place and place attachment, both of which can play a beneficial role in psychological health and well-being.

## 4. Discussion and Conclusions

This article synthesizes literature related to the ecosystem services (e.g., recreation, climate mitigation, and aesthetic value) provided by urban green spaces. We maintain that minority and low income population engagement with and proximity to green spaces may reduce health disparities in obesity, cardiovascular and heat-related illness, and psychological concerns. Encouraging equitable access to green spaces is a key step in promoting environmental justice [124]. However, as discussed, the type, quantity, condition, biodiversity, and overall distribution of green spaces can fluctuate throughout a given landscape and influence the ecosystem services ultimately received [27].

Research in public health and environmental conservation should aim for policy driven projects that pose practical questions related to the natural environment and its role in health promotion [25], especially in racially/ethnically diverse and socioeconomically disadvantaged communities. A great amount of research in the leisure sciences and outdoor recreation fields has examined questions related to the relative lack of outdoor recreation participation by racial and ethnic minorities, compared to whites [125]. However, engaging diverse populations in the outdoors is not only an issue for the recreation and environmental stewardship scholarship but also public health. The multidimensional potential of green spaces for minority and low-income population health has not been fully explored. Federal agencies and non-profit organizations recognize this link and have built strategic collaborations to address mutual concerns [126]. For example, the 21st Century Conservation Corp supports programs to engage and employ young people, including those from underserved communities, in outdoor recreation and conservation programs [127]. Organizations like Girl Trek have partnered with the National Park Service to promote physical activity amongst black women and increase park visitation [128]. Similarly, the Forest Service funds numerous “Kids in the Woods” projects that encourage environmental education and physical activity in communities across the nation. Future research can analyze the health implications of such projects.

### Moving Ahead

Although we argue that the uneven distribution of ecosystem services can impact health, caution must be taken to not exaggerate the relevance of green spaces over other major factors involved in health disparities, such as access to health care, education, systematic/institutional barriers, and environmental burdens. Indeed, the efficacy of green spaces in ameliorating health conditions is dependent upon the stable or positive condition of these and other crucial aspects of place. Also, the effectiveness of green spaces in redressing health disparities is contingent upon the nature of illness and its interaction with various ecosystem services supported by that vegetation [129]. Certainly, green spaces can be linked to “disservices” that may be adverse to public health such as pollen (e.g., prompts allergies or worsens respiratory conditions), habitat for insects that carry vector diseases [130,131], or storm damage [132]. This is an important caveat in the “green space/health” discussion but is beyond the scope of this article. Future work on this topic should account for both the real and perceived negatives associated with urban greening. Methodological challenges involved with studying the intersection of green space and health include: measuring exposure without assuming that the study population interacts with nature in a way that is beneficial to health, quantifying outcomes, establishing a causal relationship (not merely associative) at the population level, and understanding the underlying mechanisms (e.g., exposure pathway) [8,19,42].

Also important to this topic is the influence of population density, that is, whether the populations examined are urban, suburban, or rural. This paper focuses on the benefits of urban green space to urban residents, but green spaces in a larger sense (i.e., national parks and forests, privately-held woodlands) are also abundant in rural settings and often overlap disadvantaged populations. For instance, persistent poverty exists in rural areas of the U.S., especially in counties across the South and Southwest that are dominated by blacks and Hispanics, or are near Native American lands, while impoverished majority white areas cluster in Appalachia [133]. We are aware of only one study that examined the relationship between green space and health in a rural context [115]. That study was conducted in Wisconsin, where rural conditions, we would argue, are qualitatively different than those in the rural South. The lack of rural-based studies on this topic is likely due to the preponderance of global populations in urban areas and the difficulty controlling for factors that influence health outcomes (e.g., access to doctors, transportation, and education, among others). However, if the goal of future research is to produce more nuanced examinations of health disparities related to the presence or absence of green spaces, then it is imperative to consider this question in all places where ethnic/racial minorities and lower income populations predominate. We recommend that the literature on green space and health would be enhanced with studies addressing rural minority and low-income populations. In addition, we suggest six other areas for future research on green space and racial/ethnic health disparities:

- Examine how ecosystem services, especially those related to social interactions, physical activity, and climate adaptation, are being assessed in disadvantaged communities,
- Determine the effect of “park prescriptions,” that is, a prescription from a medical professional directing more time spent in the natural environment, on cardiovascular and psychological health outcomes,
- Examine the role of green space programing on sense of place,
- Investigate how changes to green space influence the level of outdoor physical activity,
- Analyze how changes in green space relate to local temperatures and social relations,
- Explore how the distribution of green infrastructure is linked to water quality in disadvantaged communities

Other recommendations to minimize some health disparities linked to urban green spaces include: improve safety in disadvantaged communities, support municipal projects to strategically plant and maintain urban green space projects, include urban ecologist/landscape architects and arborists in the planning process, promote programming and social events on green spaces, and audit the coverage and condition of green spaces to support climate adaptation goals. It can be challenging to apply the knowledge we already have when fields such as urban ecology and public health interact as acquaintances instead of partners working toward a mutual goal of overall societal well-being. Transdisciplinary research is needed to bridge these divides. Similarly, it is imperative that the vision of “biophilic cities,” which integrates nature into urban planning, [134] reflects the principles of environmental justice that can help reduce health disparities. We hope that this article stimulates collaboration and helps to leverage resources to collectively address environmental health disparities as they relate to ecosystem services from green spaces.
